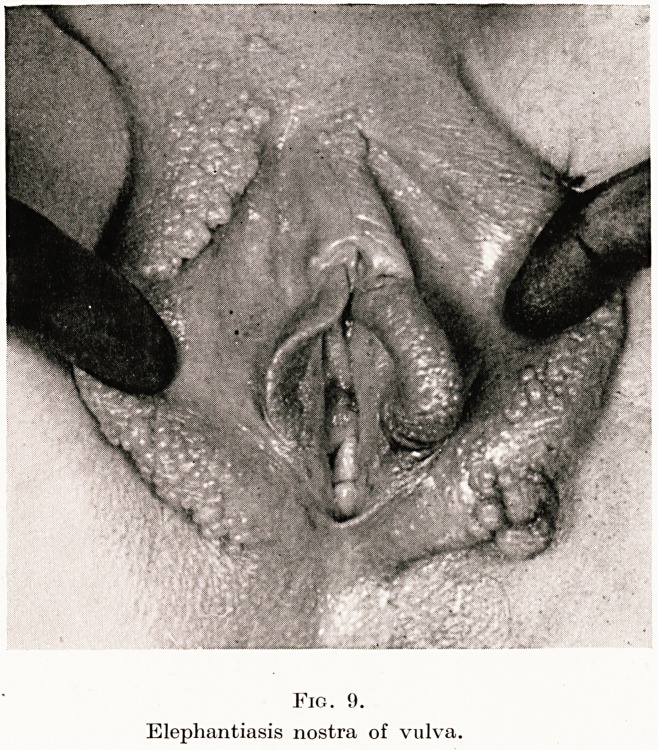# The Dramatic in Gynæcology and Obstetrics
*An address delivered to the Bristol Medico-Chirurgical Society on Wednesday, 9th February, 1949.


**Published:** 1949-07

**Authors:** V. B. Green-Armytage

**Affiliations:** Consultant Gynæcologist, West London Hospital and British Post Graduate Hospital and Hospital for Tropical Diseases, London


### PLATE IX

FIG. 1.
Osteomalacic deformity.

FIG. 2.
Osteomalacic deformity. Arm, leg and pelvis after Caesarean.

FIG. 3.
Osteomalacia, showing triradiate deformity of brim, contracted outlet and osteoporosis.

### THE DRAMATIC IN GYNÆCOLOGY AND OBSTETRICS*

BY

V. B. GREEN-ARMYTAGE, M.D., F.R.C.P., F.R.C.O.G.

*Consultant Gynæcologist, West London Hospital and British Post Graduate Hospital and Hospital for Tropical Diseases, London.*

THE *Oxford Dictionary* defines drama as "a set of events having the unity and progress of a play, leading to catastrophe or consummation". That being so, I am very conscious of the fact that in my career the dramatic faced me early. It was in 1904 when I was on the district all by myself in that unsavoury part of Bristol which used to exist to the right and left of those interminable steps which lead directly north opposite the Royal Infirmary. In those days there was no antenatal care and the "doctor" was only called when labour had begun. I remember I had been up all night waiting plaintively for the high bulging membranes to rupture. At last as dawn was breaking I plucked up courage and despatched a greasy note by the poor husband to Dr. Stack the R.O.O. He wrote back on the note "rupture them yer fool!" As my fingers slipped and slithered, being unable to scratch their way through the membrane, I used a pair of scissors. Out gushed brain and liquor cerebri in vast quantities. It was a case of hydrocephalus!

Since then I have worked and wandered in many lands. Working in this country one tends to forget the frightful deformities of osteomalacia and the skill necessary to deliver such patients. Indeed, I think that few of us realize the grand work that is done by missionary doctors and civil surgeons in many parts of the Empire. I do not speak only of a nightmare craniotomy through an outlet of perhaps only two inches, but rather of the Caesarean where owing to fixity and flexion of the thighs, the lower segment cannot be reached and the only portion available, or for that matter, visible, is the fundus. You may well imagine what tragedies and injuries occur and what the drama of a live childbirth is to all concerned. (Figures 1, 2, 3.)

Another urgent problem of the East is the flabby abdominal wall with a flabby uterine wall and a flabby cardiac wall, which so

* An address delivered to the Bristol Medico-Chirurgical Society on Wednesday, 9th February, 1949.

often complicate labour and terminate in tragedy. I recall one such patient, an Englishwoman, who had not only the typical pendulous belly of the Orient, but also an umbilical hernia. (Figure 4.) Despite all care to prevent disaster, this patient died of shock immediately following acute inversion, although the attendant knew full well what might occur and did his utmost to avert catastrophe.

I shall show you slides of Elephantiasis vulvae, a condition also quite commonly seen to-day in the West Indies, and a radiograph of the foetus *in utero*, demonstrates the high degree of moulding which had occurred after death of the foetus.

There have been times when literally even my scanty hair has almost stood on end. For instance, I recall an incident when I was applying forceps and not difficult ones at that, when suddenly I heard a sound like the crack of a pistol; the symphysis had ruptured and one could put two fingers between the pubic bones. Mother and child did well, however.

I remember another occasion when I was called by a doctor at dead of night to a patient's house to apply forceps. The lady had been in labour a long time and was lying on one of those huge, soft, sagging double beds. I placed her buttocks over the hard edge of the bed, not at the time realizing that the rest of her body was almost in the Trendelenberg position. As I adjusted the forceps, the foetus uttered a prolonged wail, identical in every way to that of a new-born babe—a wail that was plainly heard by everybody in the room, and before I could deliver that child, this awe-inspiring, hair-raising, vagitus uterinus was repeated four times. The infant was born alive and did well.

Another horribly dramatic experience, one that must have occurred to some here, was when I heard the humerus crack while my resident lady doctor was bringing down an extended arm in a difficult breech presentation.

*Opéra bouffe* has occasionally come my way. For instance a few years ago I was asked to see a case of supposed hydatidiform mole. The uterus was considerably larger than it should have been for its dates and there was a slight sanious discharge. As the uterus was up to the umbilicus and no foetal heart sounds could be heard, I suggested that an X-ray might help. No foetus was visible, but as you will see, a Graffenberg ring was jauntily embedded in the placenta. (Figure 5.) The patient went to term with twins: and when the silver ring was dug out afterwards from the placenta, she wore it as a brooch for many years!

I remember being called to a nursing home in Chingford in Essex one cold, foggy night. The Irish doctor on the 'phone said it was a case of acute hydraminos and that the patient was very ill. When seen she presented a tense, shiny, distended abdomen; the tumour was obviously fluid. On passing a catheter I let out fourteen pints

PLATE X

Fig. 4.
Pendulous belly with umbilical hernia in primipara—subsequent inversion and death.

Fig. 5.
Graffenberg ring in situ in case of twins at fifth month.

PLATE XI

Fig. 6.
Large ovarian cyst.

Fig. 7.
Obstruction by round worm infestation simulating ovarian cyst.

of urine in the course of three hours. She went to term after replacement of the uterus and the wearing of a catheter for a week. Slow decompression is not necessary in such cases.

Coming to gynaecology, Figure 6 shows one of two cases that I saw during the bombing of London in 1941. Both were Christian Scientists and both were patients whose relatives had refused to stay with them in much "blitzed" Fulham. The removal of such colossal tumours is of course dramatic: but provided one can prevent shock, anyone who has been trained in the Tropics need have no fear, for such tumours even to-day are to be seen there every week.

Figure 7 is in a sense even more remarkable; for here the abdominal swelling is not due to tumour, pregnancy or ascites, but to chronic intestinal obstruction caused by the accumulation of roundworms by the ten thousand, a condition only to be seen in the Tropics. The effect of the treatment was indeed dramatic.

The problem of fistulae and particularly the persistent vesico-vaginal fistula, whether seen here or abroad, is a dramatic one. Who has not heard such a patient say " let me be dry or die"? Compare the breath-taking beauty of young West African pagans before marriage, and the appearance a few years later of two of these girls after a complicated labour resulting in large vesico-vaginal fistulae necessitating sigmoidal implantation of the ureters.

There is an idea prevalent here, fostered by Oxford, that all such fistulae can be cured vaginally by silver wire. Believe me, that is nonsense: for the size and edges of these holes have to be seen and felt to be believed. Consequently it is imperative that every missionary doctor or otherwise proceeding to the Tropics should be absolutely conversant with the technique of implantation of the ureters into the bowel. This operation indeed combines drama with thanksgiving.

Which of you has not seen the high recto-vaginal fistula that has defied two, three or even more attempts at closure? Only recently I had such a case sent to me. Yet if the technique be changed, what melodramatic rejoicing follows success.

In 1937, I visited Alexandrov's clinic in Moscow. His operating theatre had two tables in it—the first was occupied by a patient having a myomectomy and simultaneously a cadaver blood transfusion; whilst on the other sat a tough-looking girl avidly watching the proceedings whilst awaiting her own spinal anaesthetic.

One of my most dramatic memories concerns being directed by the Foreign Office to proceed to Nepal and do a hysterectomy on a V.I.P. The journey took five days by train, elephant, foot and dandy, that is, carrying-chair, up and down the mountains. On arrival my horoscope was first carefully read and when declared lucky (for my birthday is on August 14th which means the happy conjunction of some particular stellar units) I was asked the following day to operate in the excellent public hospital of Khatmandu on two Nepalese women who presumably had the same fibromatous condition as Her Excellency. These surgical results being satisfactory, a bedroom with marble walls and floor was selected in a wing of the palace as the theatre.

The day, the hour, the minute for the laparatomy was next fixed by the soothsayer. That morning began a play reminiscent of ancient Rome. The very charming lady dressed in a new white silken sari with her hair decked with sprigs of fresh corn and rice, and most conspicuous of all, a banana, supported by two female attendants, walked slowly down a long white marble corridor lined by a host of her retainers to whom she threw as largesse, gold, silver and copper coins. Her husband and I with my theatre staff very humbly followed behind. She showed no signs of fear. Throughout the operation, which was watched by a hundred pair of eyes glued to the jhilmills and arabesque slots in the walls, one heard guttural wah! wahs! It was an endometrioma. I was paid in a leather sackful of silver rupees which even the elephant eyed askance when put upon its back!

Even to-day tragi-comic incidents occur. For instance, not so very long ago I saw a well-known actress in consultation who had had an emergency laparotomy on the diagnosis of a twisted ovarian cyst. Nothing, however, was found. A few weeks later after she had gone back to the stage, it was realized from her gums that her recurrent acute abdominal colic was due to lead which was being absorbed from her 'make-up'.

Quite recently another comedy came my way. An ex-proconsul from India had married a very charming girl who, because she was a bad diabetic and insulin resistant, had been told by expert physicians never to risk pregnancy. She was therefore sent to the Highest Priestess of Lady Doctor Cap Fitters. Six months later I was asked to see her. She was four months pregnant and on examination I found she had two perfect vaginae and two perfect uteri. Over the cervix of one was a metal cap! The husband had gone the other way. The High Priestess had missed the double doors. She was not amused when a lawsuit for damages was on the tapis. I did a Caesarean at full term and got a live baby. A few months later I persuaded the lady to have the hysterogram done which I show you (Figure 8).

As you know, prolapse is the bane of every Outpatient Department, but I doubt if we here outdo the East in this respect, for there it is often complicated by prolapse of the anus. It is not my intention to pause over the surgical side of such a problem, but I want to remind you that the ancient Egyptians knew all about prolapse. When the perineum was moderately intact, they employed hollow ball-like pessaries made of clay from the delta of the Nile.

PLATE XII

FIG. 8.
Double uterus. Double vagina.

PLATE XIII

Fig. 9.
Elephantiasis nostra of vulva.

I thought you might like to see the types of pessary used by these small women which were actually removed from the bodies of mummies. To me this is peculiarly interesting, for I have seen such supports *in situ*, worn by women living in Baghdad and in the valley of the Ganges. To-day if the perineum is good and a cystocele is the main discomfort, a ping-pong or rubber ball are excellent palliatives.

Even in the use of lipiodol, dramatic moments occur. I shall not lightly forget the appearance of the first salpingogram that I ever did in 1925, when by mistake I removed the nozzle of the syringe before the picture was taken; nor need I remind you of the dramatic importance of this method of investigation when there is a history of miscarriages. I show you the double uterus of a distinguished doctor's wife who had had four miscarriages for which no cause whatever could be found until the radiograph showed it. Occasionally of course one has the alarms and excursions of oil embolism and perforation, but they are very rare and *not* dangerous like air embolism with its tragic deaths on the table, that is if you should still use air rather than carbon dioxide. I have had just such a death and can never forget it, and recently there was another in London.

Some may think that nowadays we are so advanced that the old-fashioned disasters no longer occur. Let me tell you of a case that arrived by air quite recently from Singapore. The lady had had a live baby with some forceps difficulty. Three days later she developed general peritonitis for which the abdomen was twice opened and drained. She recovered: but pus continued to pour from the wound for eight months. Suddenly one day hairs were seen in the pus—it was at once realized that the foetal head had compressed and burst a dermoid cyst of the ovary. She was then sent home by air. That cyst was the size of a tennis ball, deep down in the pelvis and surrounded by matted coils of bowel. I found the removal a nightmare! You can see the teeth outlined in the lipiodologram.

Figure 9 shows a case of elephantiasis nostra which occurred in the wife of a Wessex dentist. She was twenty-six and had never been out of this country. Vulvectomy was performed necessitating Caesarean section the following year—a set of events, as the definition goes, having the unity of a play leading to consummation.

## Figures and Tables

**Fig. 1. f1:**
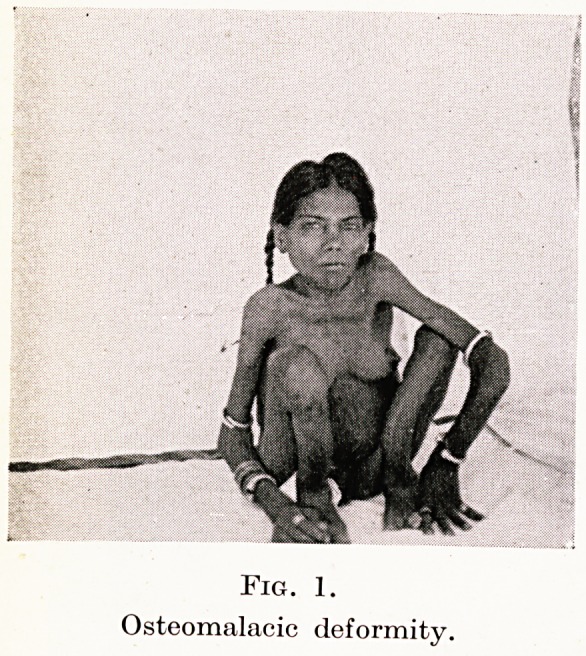


**Fig. 2. f2:**
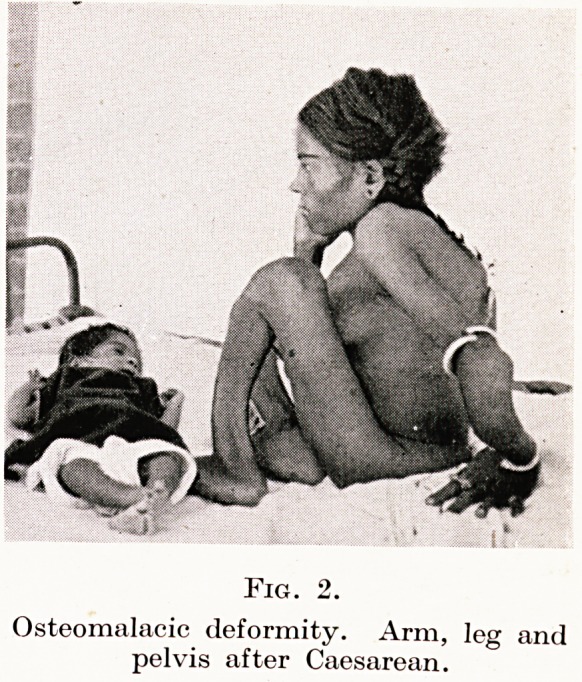


**Fig. 3. f3:**
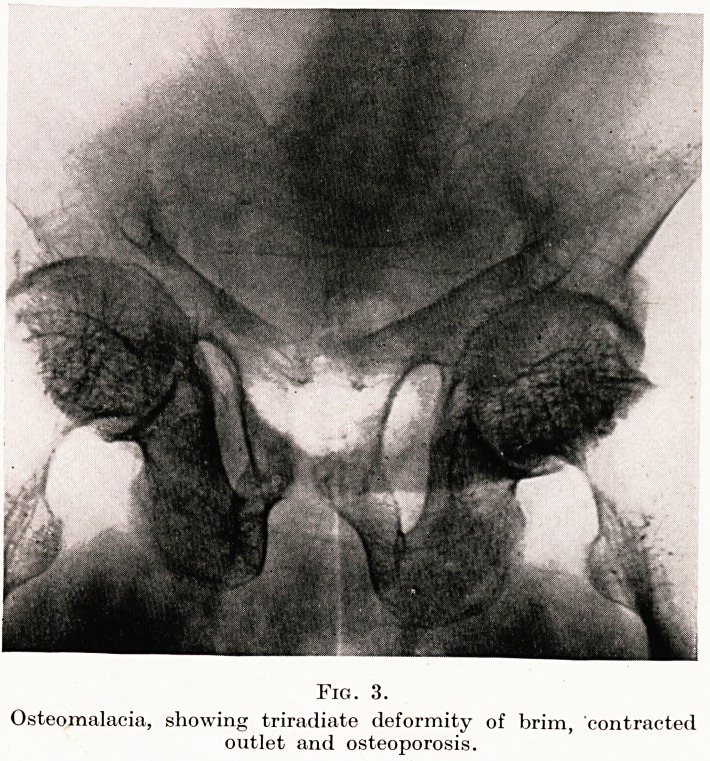


**Fig. 4. f4:**
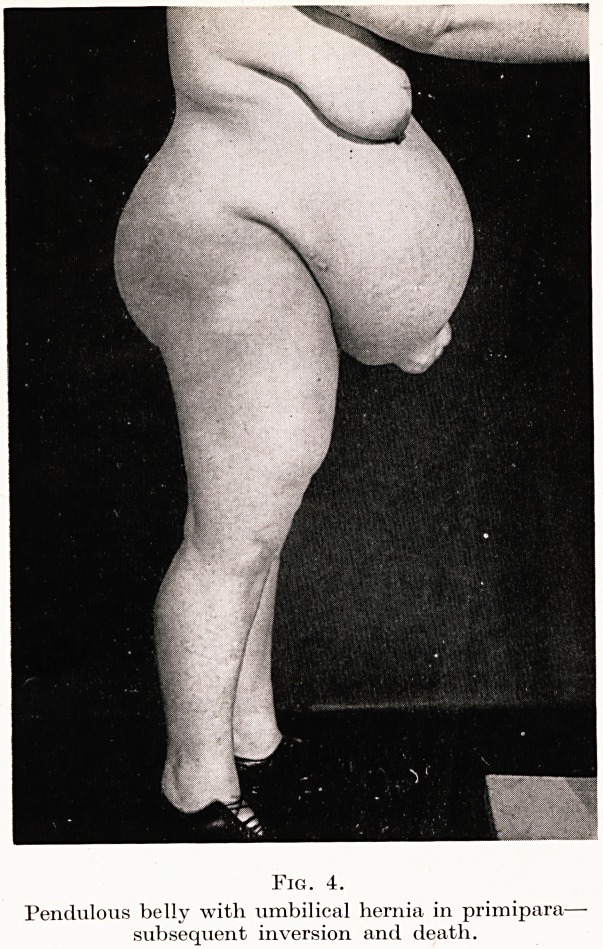


**Fig. 5. f5:**
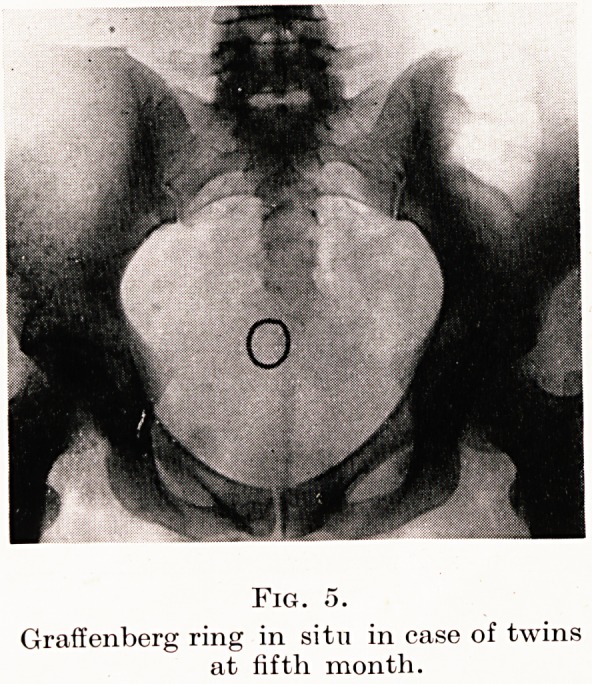


**Fig. 6. f6:**
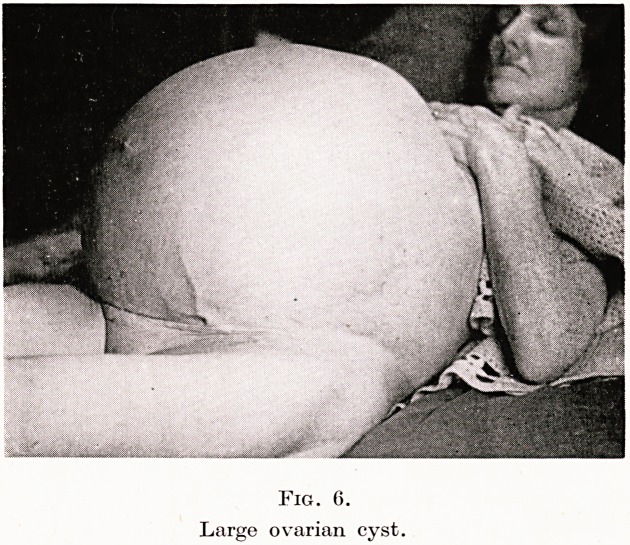


**Fig. 7. f7:**
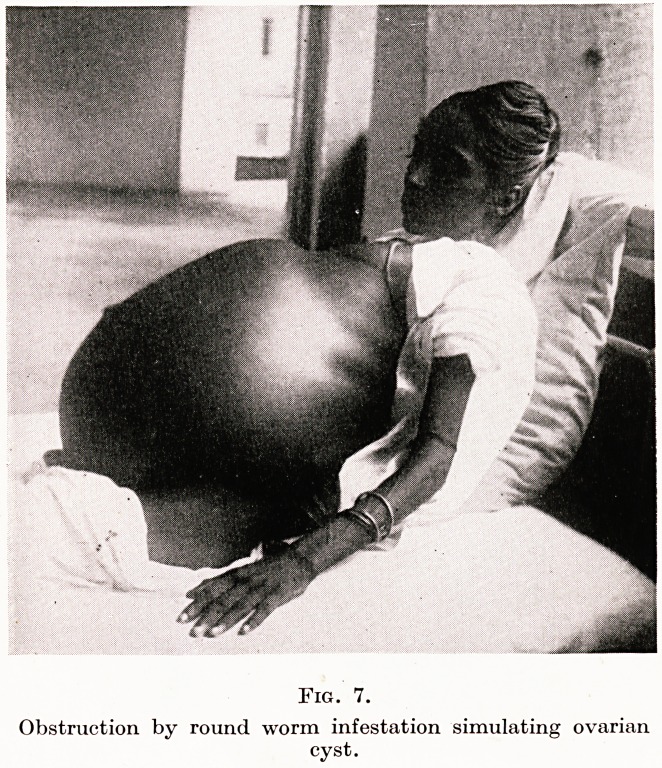


**Fig. 8. f8:**
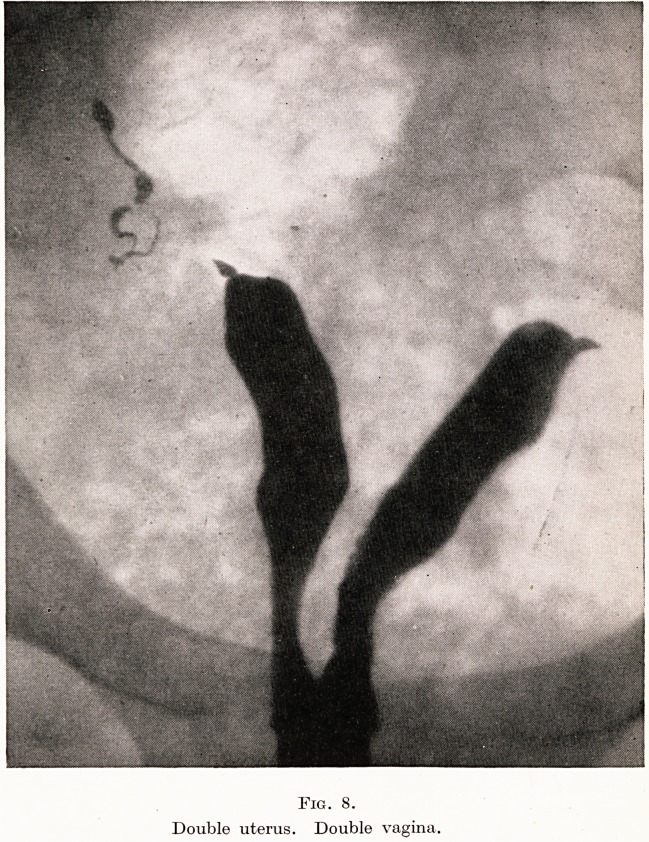


**Fig. 9. f9:**